# Study on human subjects – influence of stress and alcohol in simulated traffic situations

**DOI:** 10.12688/openreseurope.13592.1

**Published:** 2021-07-27

**Authors:** Mobyen Uddin Ahmed, Mir Riyanul Islam, Shaibal Barua, Bertil Hök, Emma Jonforsen, Shahina Begum

**Affiliations:** 1 School of Innovation, Design and Engineering, Mälardalen University, Västerås, Sweden; 2 Senseair AB, Flottiljgatan 49, SE-72131 Västerås, Sweden

**Keywords:** SIMUSAFE, Study on Human Subjects, Influence of Stress and Alcohol, Simulated Traffic Situations

## Abstract

This report presents a research study plan on human subjects – the influence of stress and alcohol in simulated traffic situations under an H2020 project named SIMUSAFE. This research study focuses on road-users’, i.e., car drivers, motorcyclists, bicyclists and pedestrians, behaviour in relation to retrospective studies, where interaction between the users are considered. Here, the study includes sample size, inclusion/exclusion criteria, detailed study plan, protocols, potential test scenarios and all related ethical issues. The study plan has been included in a national ethics application and received approval for implementation.

## Introduction

### Project overview

The SIMUSAFE project (an acronym for “SIMUlator of behavioural aspects for SAFEr transport”) aims to analyse and define the individual variable related to risky uptake behaviour in urban traffic situations, trace cause-consequence data to evaluate risk awareness and perception and determine core factors of risky behaviour and affected decision-making processes. SIMUSAFE therefore makes use of state-of-the-art simulation, artificial intelligence (AI), virtual reality (VR) and data science methodologies to retrieve accurate actor and behavioural models in the urban traffic environment, so as to reproduce the same circumstances in incidents of interest, and thus to understand the underlying behaviour and motivations of the involved actors.

For the present human subject study, simulators have been prepared for the following scenarios and road-users:

- Car drivers
- Motorcyclists
- Bicyclists
- Pedestrians

**Figure 1.**
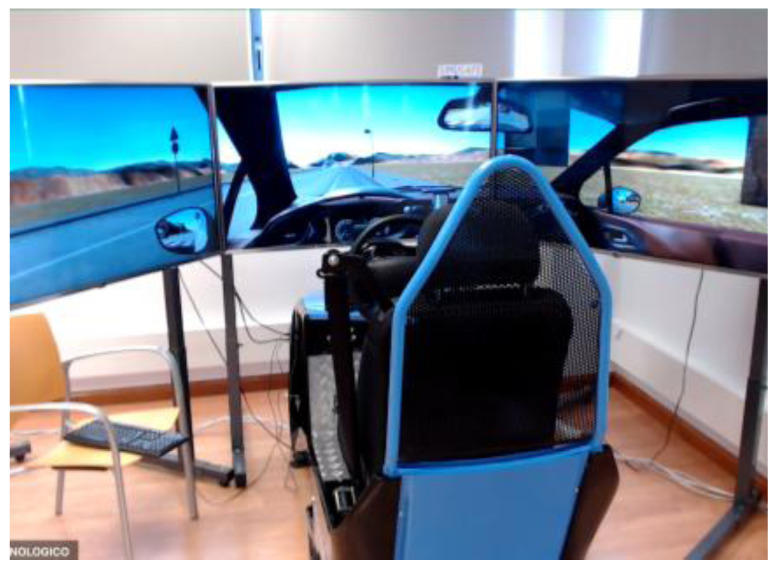
Car Simulator.

In Figure 1– Figure 4, pictures (taken by ITCL) of the car, motorcycle, bicycle and pedestrian simulators are shown. The vehicle simulators include big screens and pedestrian simulators include VR goggles that simulate the specific conditions of each category.

**Figure 2.**
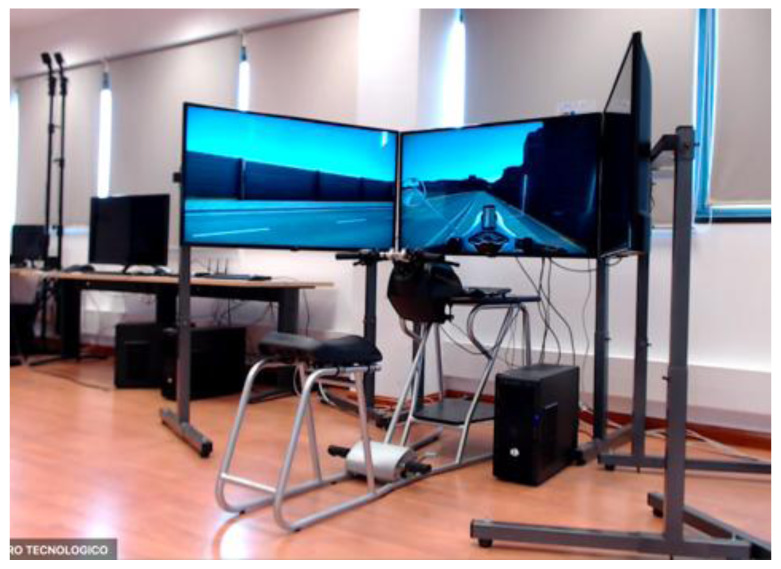
Motorcycle Simulator.

**Figure 3.**
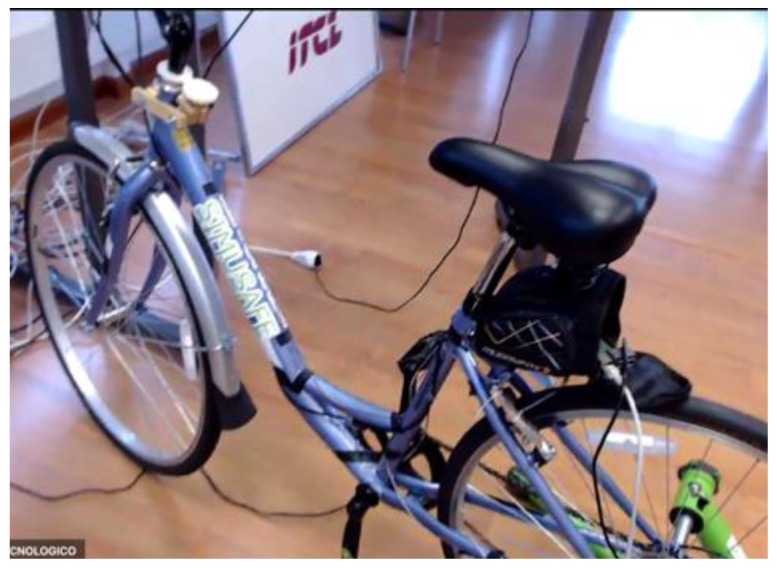
Bicycle Simulator.

**Figure 4.**
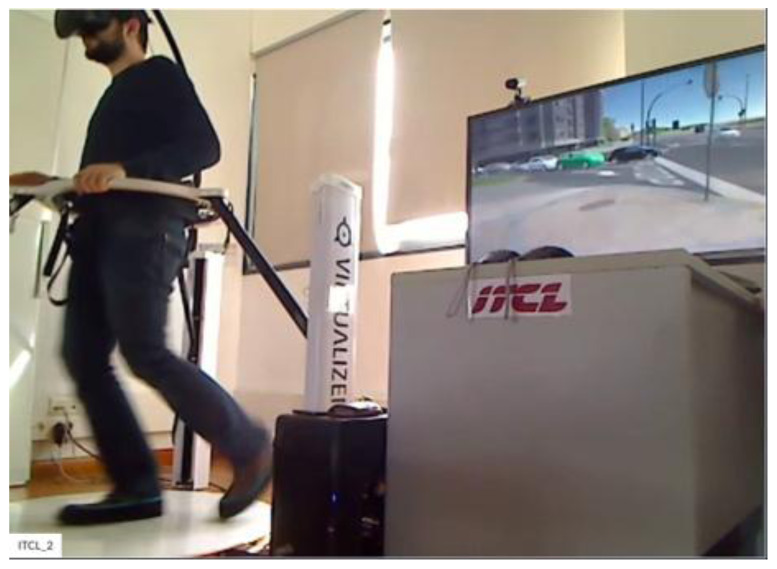
Pedestrian Simulator.

### Background

This phase of the SIMUSAFE project is focused on a major issue, stress, which affects a road user’s behaviour while actively participating in regular traffic. The growing complexity of the car and traffic environment, including the operation of assistance and information systems, can induce stress and increases cognitive load, which may affect driving performance ^1^. Mathews & Desmond outlined two major causes of driver stress: overload of attention and disruption of control ^2^. Driving environment can relate to both aspects. For example, road condition, such as road that causes excessive vibration to the vehicle, can overload driver attention and overall workload of the driver increases. This phenomenon can produce increased stress for drivers. Moreover, the environment of the road equally affects a pedestrian’s stress level while using the road. Selzer & Vinokur outlined driver stress as the prime safety problem for road users since it provides insights on driver behaviour by defining cognitive load ^3^. Several studies were conducted to determine the effects of stress on driving performances ^4, 5^. Most of these studies found that driver stress impairs driving performance and increases risk-taking behaviour to cope with stress. Risky behaviours further stimulate aggressiveness in driving performance and end up in severe crashes that harm other normal road users ^6, 7^.

The influence of alcohol intoxication on human behaviour in relation to traffic safety has been extensively studied over several decades. This research has been strongly motivated by the fact that a small fraction of drunk drivers, less than 1% in most countries, typically cause 25–30% of road accident fatalities ^8^. Legal limits for breath and blood alcohol concentration have been established in most countries based on physiological research establishing the blood/breath transition, which Jones and Hlastala showed in their studies ^9, 10^. In an extensive retrospective case-study by Blomberg *et al.* ^11^, it was established that the accident risk increases rapidly and progressively with alcohol concentration. The results are summarized in Table 1, showing that below the Swedish breath alcohol concentration (BrAC) legal limit of 0.1 mg/L, the risk increase is only a few percent. In the interval of moderate intoxication close to the legal limit in most EU countries (0.2–0.3 mg/L), the risk increase is considerable, 18–69%, whereas in the interval 0.4–0.6 mg/L of high intoxication, the risk increase is several hundred percent.

**Table 1.** Influence of breath alcohol concentration (BrAC, mg/L) on relative accident risk, based on the publication ^11^.

Category	BrAC Interval (mg/L)	Relative Accident Risk Interval
Sober	0-0.1	1.00-1.03
Moderately intoxicated	0.2-0.3	1.18-1.69
Highly intoxicated	0.4-0.6	2.69-8.90

In recent years the technology for contactless breath alcohol analysis has evolved ^12, 13^, raising the feasibility and awareness of new solutions for passive in-vehicle detection ^14, 15^.

Simulators for vehicle driving have been known for decades, but to our knowledge no studies have been performed so far to investigate the influence of stress and alcohol intake on driver performance in a simulated environment, and to compare these results with other study results.

The potential benefits of advanced simulators should be obvious both in research and for educational purposes. The possibility to assess and improve behaviour could potentially improve traffic safety, saving lives and preventing injuries and damage related to traffic accidents.

### Study objective

The main study objective is to evaluate the performance of the simulators with respect to the influence of stress and alcohol consumption on road-user behaviour in relation to retrospective studies.

The following research questions are raised in the study:

i. How do different road conditions and situations impact the driver’s behaviour? Is the driver’s behaviour different with respect to the same conditions/situations if the driver is experiencing stress? Does the physiological stress change the risk potential of specific situations?
ii. Alcohol related question: Is there any correspondence between the behavioural results of the study and the dependency of accident risk on alcohol concentration according to retrospective studies, e. g. Blomberg *et al.* ^11^?
iii. Are there any unexpected results from the study?

## Protocol

### Selection of participants

A total number of 90 participants will be recruited for the study on the overlap between stress and alcohol. The selection will be made from a larger number of people who will be asked to fill out a questionnaire, and who will also be interviewed to establish their qualifications to be enrolled as participants. The related information sheet, consent form and questionnaire are provided as *Extended data* ^16^.

Each recruited participant will be called to the test site on two occasions, once for stress-related tests and once for alcohol-related tests. The test subjects are divided into two age groups: young (18 – 25 years) and elderly (58 – 70 years). Participants for the stress test will receive a gift card of worth 1500 SEK as gratification for successfully completing all stages of the test. For alcohol test participants the amount of gratification is 2000 SEK. Table 2 and Table 3 demonstrate the approximate number of participants from different age groups and road user types, respectively.

**Table 2.** Number of subjects from different age groups.

Tests	Young adult	Elderly	Total
*Alcohol*	20	25	45
*Stress*	20	25	45
**Total**	40	50	90

**Table 3.** Number of participants from different road user types.

Actors	Young adult	Elderly	Total
*Car drivers*	5	7	12
*Motorcyclists*	5	6	11
*Cyclists*	5	6	11
*Pedestrians*	5	6	11
**Total**	20	25	45

Being the first investigation of its kind, this study is exploratory rather than confirmatory (i.e., a hypothesis test). Therefore, it is not meaningful to perform a statistical power analysis. The size of the study population has been established from the estimated division into age groups and other category divisions with the purpose of obtaining exploratory quantitative results.

Inclusion criteria for the participants are:

- Age: young adults (18 – 24 years) and elderly (50 – 70 years)
- Gender: male or female
- Driving license: yes (for car drivers and motorcyclists)
- Health/medicine: none (i.e., participant shouldn’t have any health related problem e.g. diseases and not be taking any medication)
- Language: English or Swedish
- Body height: 160 – 210 cm
- Body weight: 60 – 150 kg
- Alcohol usage: 5 –15 glasses per month (applicable for alcohol test only)
- Stress tolerance: only subjects with standard or high scores in the Vienna test measuring stress tolerance and flexibility.

Exclusion criteria for participant selection are motion sickness, problems using VR goggles, stress intolerance, hearing or vision impairment, presence of chronic disease or serious allergies and any relation such as teacher and student or employer and employee among the participants and researchers. Habitual computer games players will be excluded if they are expected to be “immune” to simulated stressful situations.

Based on the study plan a press release named ‘ Smarter AI vehicles with the help of citizens in Västerås’ has been posted on the Mälardalen University website. In the press release there is a Google form link that the participants can fill in and an email address for direct contact if they wish to participate in the study.

### Platform

Simulators for the concerned tests will be provided by one of the SIMUSAFE partner institutions ITCL CENTRO TECNOLOGICO, Spain. Figure 1 demonstrates the simulator for car drivers. It has three LCD displays for visualization of the frontal view with a horizontal field of view of 120 degrees, steering wheel and driver’s seat with proper safety measures. Simulators for motorcyclists and cyclists are depicted in Figure 2 and Figure 3, respectively. Participants for both motorbikes and cycles will have similar frontal views with LCD displays. Treadmill and VR accessories will be used to run the tests with pedestrians. Figure 4 shows the experimental setup for pedestrians. There will be two simulators for each type of actor in the test premise. The simulators occupy approximately 10 m ^2^ of room area and can be positioned in almost any office or laboratory environment.

### Data/statistical analysis

The data analytics will comprise a data storage infrastructure to gather all relevant data (actor model states, user, cognitive and behavioural assessment data and annotations). This infrastructure will be integrated in IBM Bluemix cloud platform together with the Sensorization Platform, further processing will be performed in the Data Analysis Server. IBM Streaming and Predictive Analytics from the platform will be employed to pre-filtering raw data sent to the Data Analysis server, for the real-time identification of events of interest and characteristic data patterns to be translated into components of the Actor Model. This data analysis to obtain required performance and isolate impacting factors will operate in three phases.

Phase 1 information fusion, data abstraction, and data pre-processing will be performed based on a combination of statistical, machine leaning and signal processing methods and techniques. Here, a robust and scalable data cleaning strategy will be established with domain-specific knowledge, including sub-processes like cleaning, filtering, sampling or/and normalization. Previous work on data pre-processing ^17^ using both structured and unstructured data will serve as basis for online automated data cleaning. Traditional feature extraction methods ^18^ will be adapted to handle scalability issues in the domain. Novel strategies to fuse data at feature level and at data level considering a defined fusion mechanism ^19^ will be used.

During Phase 2 data mining and knowledge discovery, a combination of potential sequences in the learning and search procedure will be investigated. The similarity assessment in the time series will be done by measuring the distance between probability distributions in the time series data mining ^20^. A combination of statistical model and fuzzy modelling algorithm will be applied to automatic addition/deletion of rules, as well as adjustment of the membership functions. A continuous learning procedure will be developed to keep the model constantly updated ^21^. New mining methods for the discovery of knowledge will be developed.

For Phase 3 learning, reasoning and model creation, adaptation of dynamic knowledge representation approaches will be achieved by combining different artificial intelligence ^22^ methods. This has a connection with Phase 2 as the data driven knowledge, rules and patterns will be considered as input. To provide decision support, hybrid approach will be applied using different traditional machine learning algorithms, such as case-based reasoning, and clustering ^23^.

### Design and procedure

One test leader, Mobyen Uddin Ahmed from MDH, will be assigned to the management of the stress-related tests and Jonas Ljungblad from Senseair will be assigned to manage the alcohol-related tests. The detailed description of different test scenarios and procedures are described in the following sections.

***Stress test*.** The stress test will be performed as a within-participant design. To induce stress during this part while driving, the participants will be asked to perform other specific tasks where a continuous increase of cognitive load will be required. To keep cognitive loads increasing during the performance, two tasks relevant for the purpose have been selected: time pressure and social stress. During the scenarios, three levels of stress will be induced to the participants. These levels are numbered as 0, 1 and 2. Stress condition with level 0 will be considered as the baseline. Consequently, levels 1 and 2 will be considered as low and high stress, respectively. The low stress condition will include time pressure and the high stress condition will include time pressure and social pressure. Moreover, the participants will drive on three kinds of road (driving condition) and a limited number of events and hazards will occur, allowing study of the drivers’ risk perception and behaviour when coping with unexpected risky events under increased cognitive load conditions. For bicyclists there will be only two types of driving condition. From the total number of participants, approximately 12 will be taking part in this test and they will be divided into three groups to randomize the sequence of stress-eliciting factor insertion during driving or riding a motorcycle in the simulation. For cyclists there will be only two subject groups containing approximately 22 participants each. Description of scenarios and the randomized sequence of scenarios in simulators for different actors are given in the subsequent sections below. Table 4 illustrates the sequence of induction of stress to participants for 32 minutes of simulator participation. The time for simulator usage will differ for different actor groups. For cyclists, scenario S0.3 and S1.3c will be replaced by similar scenarios defined below. The notations for different scenarios in Table 4 are further defined in the subsequent sections for different actor groups.

**Table 4.** Test order for the different actors for stress.

Time	4	4	4	4	4	4	4	4	Total
**SG – 1** **DC – 1**	S1.0 + S2.0 (BL)	S1.1 (LS)	S1.1 (LS) + E1.a	S1.1 (LS) + E1.b	S1.0 + S2.0 (BL)	S1.1 + S2.1 (HS)	S1.1 + S2.1 (HS) + E1.b	S1.1 + S2.1 (HS) + E1.c	**32** **mins**
**SG – 2** **DC – 2**	S1.0 + S2.0 (BL)	S1.1 (LS)	S1.1 (LS) + E2.a	S1.1 (LS) + E2.b	S1.0 + S2.0 (BL)	S1.1 + S2.1 (HS)	S1.1 + S2.1 (HS) + E2.b	S1.1 + S2.1 (HS) + E2.c	
**SG – 3** **DC – 3**	S1.0 + S2.0 (BL)	S1.1 (LS)	S1.1 (LS) + E3.a	S1.1 (LS) + E3.b	S1.0 + S2.0 (BL)	S1.1 + S2.1 (HS)	S1.1 + S2.1 (HS) + E3.b	S1.1 + S2.1 (HS) + E3.c	

SG: subject group, DC: driving condition, BL: baseline, LS: low stress, HS: high stress.

During the experiments, two participants from an actor group will be tested in the simulator each day. Each of the participants will be clearly briefed about the task and their responsibilities. Afterwards, physiological equipment will be attached to their body. Each participant will require around 2.5 hours to prepare fully for the tests. They will participate for about 40 minutes including some practice. After the test finishes, they will be debriefed, and the equipment will be removed. Table 5 shows the time plan of a test day for participants (car drivers, motorcyclists and cyclists).

**Table 5.** Schedule of a test day for participants in stress test.

Time	Test events
**Participant 1**	
08:00	Arrival at Senseair
08:10 – 08:20	Primary training
08:20 – 10:00	Preparation of physiological equipment
10:00 – 10:30	Training on levels of stress & scenarios
10:30 – 11:30	Performing in simulator + practice
11:30 – 11:50	Removal of physiological equipment and debriefing
**Participant 2**	
13:00	Arrival at Senseair
13:10 – 13:20	Primary training
13:20 – 15:00	Preparation of physiological equipment
15:00 – 15:30	Training on levels of stress & scenarios
15:30 – 16:30	Performing in simulator + practice
16:30 – 16:50	Removal of physiological equipment and debriefing

*Car driver*

Different scenarios to support the levels of stress for car drivers are given below:

Driving condition – kind of road

DC – 1: City road
DC – 2: Country road
DC – 3: Highway

S1 – Time pressure

S1.0 – No time pressure
S1.1 – Time pressure

S2 – Social pressure

S2.0 – No social pressure
S2.1 – Social pressure

E – Unexpected risky events

E1 – Unexpected risky events on city road
  E1.a – Rush hour heavy traffic
  E1.b – Pedestrian crossing the road without zebra crossing
  E1.c – Diversion due to construction works

E2 – Unexpected risky events on country road
  E2.a – Sudden entry of a car from the side road
  E2.b – Motorcyclist riding in front in zigzag fashion
  E2.c – Sharp turn after an uphill and an oncoming vehicle in the opposite direction

E3 – Unexpected risky events on highway
  E3.a – Diversion due to construction works
  E3.b – Heavy vehicle entering the highway from acceleration lane
  E3.c – Low visibility due to fog

*Motorcyclist*

Different scenarios to support the levels of stress for motorcyclists are given below:

Driving condition – kind of road

DC – 1: City road
DC – 2: Country road

S1 – Time pressure

S1.0 – No time pressure
S1.1 – Time pressure

S2 – Social pressure

S2.0 – No social pressure
S2.1 – Social pressure

E – Unexpected risky events

E1 – unexpected risky events on city road
  E1.a – Rush hour heavy traffic
  E1.b – Pedestrian crossing the road without zebra crossing
  E1.c – Diversion due to construction works

E2 – Unexpected risky events on country road
  E2.a – Sudden entry of a car from the side road
  E2.b – Car driving in front in zigzag fashion
  E2.c – Sharp turn after an uphill and an oncoming vehicle in the opposite direction

E3 – Unexpected risky events on highway
  E3.a – Car cutting-in in front of the motorcycle
  E.3b – Heavy vehicle entering the highway from acceleration lane
  E.3c – Low visibility due to fog

*Cyclist*

Scenarios to be faced by cyclists in the simulator test for stress are outlined below:

Driving condition – kind of road

DC – 1: City road
DC – 2: Country road

S1 – Time pressure

S1.0 – No time pressure
S1.1 – Time pressure

S2 – Social pressure

S2.0 – No social pressure
S2.1 – Social pressure

E – Unexpected risky events

E1 – unexpected risky events on city road
  E1.a – Rush hour heavy traffic
  E1.b – Car cutting-in in front of the cycle
  E1.c – Diversion due to construction works

E2 – Unexpected risky events on country road
  E2.a – Sudden entry of a car from the side road
  E2.b – Car brushing while passing the bicycle
  E2.c – Low visibility due to dusk

*Pedestrian*

As the pedestrian simulator tends to easily cause fatigue and nausea, tests will be conducted for limited duration and will include breaks every 5 – 10 minutes. The experiments will be performed using an urban scenario with/without time and social pressure. Moreover, some particular conditions will be simulated while conducting the experiment.

S1 – Time pressure

S1.0 – No time pressure
S1.1 – Time pressure

S2 – Social pressure

S2.0 – No social pressure
S2.1 – Social pressure

C – Particular conditions

C1 – Construction on the sidewalk (requiring the pedestrian to perform a slalom)
C2 – Crowded sidewalk (lots of people moving slowly on the sidewalk)
C3 – Prolonged red light at the crosswalk (with road traffic)

Since the pedestrian simulator is the one more likely to cause discomfort, a short break time is reserved as per Table 6.

**Table 6.** Test order for pedestrians.

Time	2	1.5	1.5	3	15	2	1.5	1.5	3	Total
	S1.0 + S2.0 (BL)	S1.1 (LS)	S1.1 (LS) + C1	S1.1 (LS) + C2	Break	S1.0 + S2.0 (BL)	S1.1 + S2.1 (HS)	S1.1 + S2.1 (HS) + C2	S1.1 + S2.1 (HS) + C3	31 min

BL: baseline, LS: low stress, HS: high stress.

***Alcohol test*.** The influence of alcohol consumption on behaviour in the simulator models will be studied in each of the test subjects by first running a test in the sober condition, then consuming alcohol with 0.8 g alcohol/kg body mass. This will normally result in a breath alcohol concentration well above the US legal limit of 0.4 mg/L. A test at this intoxication level is performed in all simulators. Then the participant is allowed to rest until BrAC has declined to 0.3 mg/L, and the test procedure is repeated at this concentration level. The final test run is performed when the subject has reached below 0.1 mg/L. The decline is typically 0.08 ± 0.01 mg/L per hour ^24^ and therefore the duration of the entire test is expected to be 6–8 hours in each test subject. A tentative schedule of a test day for participants from all actor groups is given in Table 7. The exact number of simulator-driving sessions and the finish time will depend on how fast the alcohol concentration is decreasing and for how long the skin electrodes provide adequate electroencephalography (EEG) recordings.

**Table 7.** Schedule of a test day for participants in alcohol test.

Time	Test Events
**Participant 1**	
08:00	Arrival at Senseair
08:10 – 08:20	Primary training
08:20 – 09:00	Preparation of physiological equipment & practice
09:00 – 09:15	Simulator driving (sober)
09:15 – 09:45	Alcohol consumption
09:45 – 10:20	Wait to allow alcohol affecting the participant
10:20 – 10:30	Simulator driving (with high alcohol effect)
10:30 – 11:00	Wait to allow the change in alcohol level
11:00 – 11:10	Simulator driving (with decreased alcohol effect)
11:10 – 11:40	Wait to allow the change in alcohol level
11:40 – 12:00	Simulator driving (with decreased alcohol effect)
12:00 – 12:30	Wait to allow the change in alcohol level – lunch break
12:30 – 12:40	Simulator driving (with decreased alcohol effect)
12:40 – 13:10	Wait to allow the change in alcohol level
13:10 – 13:20	Simulator driving (with decreased alcohol effect)
13:20 – 16:00	Wait to diminish the effect of alcohol
16:00 – latest	Participant will be driven to his/her residence.

***Naturalistic tests in simulators*.** All of the four types of actors will take part in the simulator together to test their behaviour under the influence of stress and alcohol separately in some natural scenarios. An example task for naturalistic driving in the simulator is described below.

*Test scenario 1*

**Figure 5.**
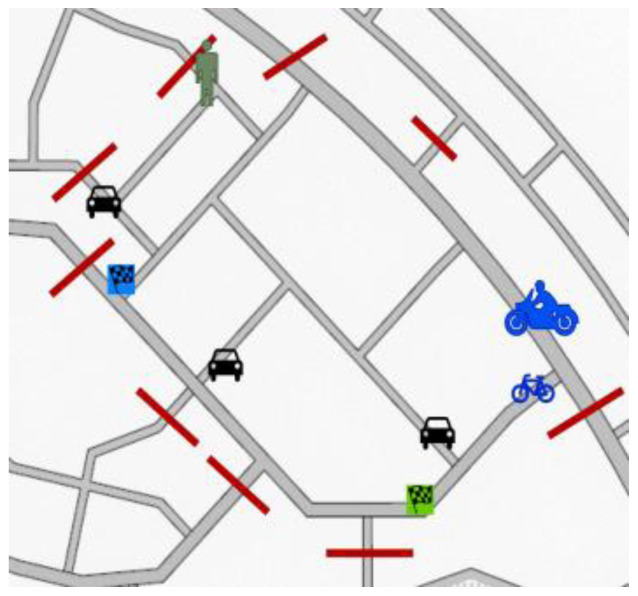
Test scenario 1.

There will be maximum of six participants taking part in the simulator study: one pedestrian, one cyclist, one motorcyclist and three car drivers. Moreover, some AI agents will be present in the simulation. Several streets in the environment will be closed due to some reasons like road or construction works etc. Participants will be participating in the test for around 5 – 10 minutes. Figure 5 illustrates test scenario 1. Each of the participants from different actor groups will have separate goals –

- Car drivers: To find a parking space quickly when only one or no parking spaces are free. The location with the unoccupied parking space won’t be revealed to the participant so they will have to drive around, as often happens in a lot of cities, to find a parking space and they will coincide on several occasions.
- Motorcyclists and cyclists: To go and come back several times between a source and destination point. It reflects a natural situation of helping a friend who is moving some stuff from an old place to a new place. Several trips are required.
- Pedestrians: Walk to a destination and return to the starting point. A friend will be waiting at the destination and the pedestrian needs to walk fast since the friend is running late for some duties.

*Test scenario 2*

**Figure 6.**
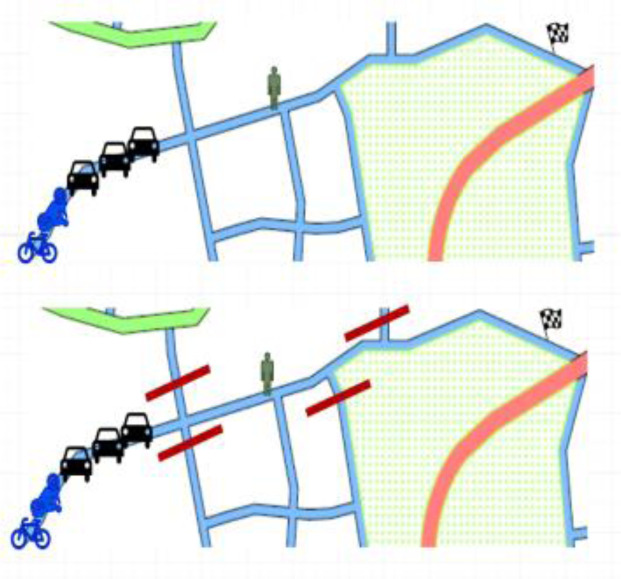
Test scenario 2.

There will be maximum of six participants taking part in the simulator study: one pedestrian, one cyclist, one motorcyclist and three car drivers. Moreover, some AI agents will be present in the simulation. Several streets in the environment will be closed due to some reasons like road or construction works etc. Participants will be participating in the test for around 5 – 10 minutes. The layout of the scenario is shown in Figure 6. Participants from different actor groups will have separate goals –

- 1st car driver: To find a parking space and to go slowly since there is only one unoccupied parking space and others behind are also looking for parking.
- Other car drivers: To reach a destination in given time for some emergency with relatives.
- Motorcyclists and cyclists: To go and come back several times between a source and destination point. It reflects a natural situation of helping a friend who is moving some stuff from an old place to a new place. Several trips are required.
- Pedestrians: To reach your destination as quick as possible; therefore, you seek the shortest route.

*Test scenario 3*

**Figure 7.**
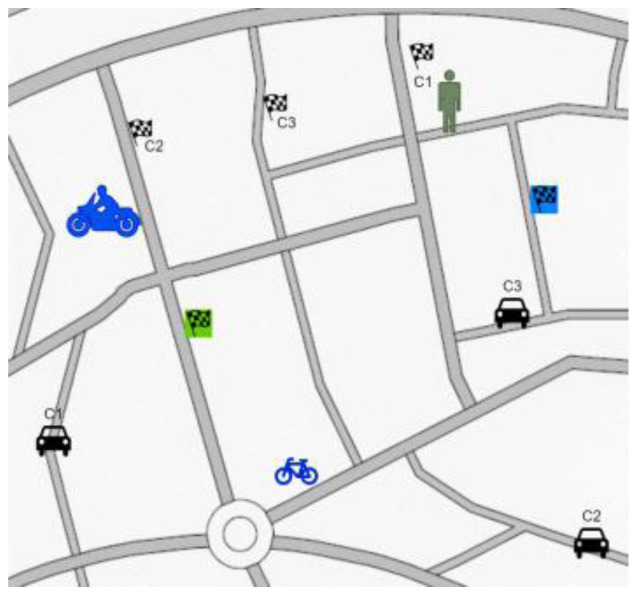
Test scenario 3.

There will be maximum of six participants taking part in the simulator study: one pedestrian, one cyclist, one motorcyclist and three car drivers. Moreover, some AI agents will be present in the simulation. Participants will be participating in the test for around 5 – 10 minutes. Figure 7 illustrates this scenario. All the participants from different actor groups will have same goal – to reach a place and come back using GPS. As the routes cross each other at several points, the users should coincide several times. The GPS will only indicate the destination with slightly different routes each time. The GPS system allows generation of routes that cross over at several intermediate points, provoking even more interactions between users.

All the participants in the above scenarios will take part in the simulating environment at the same time and will affect others’ performance as each of the difference road users do in real world scenarios.

***Coordination of stress and alcohol*.** The stress and alcohol related tests will be performed on different occasions to avoid mixing of effects.

***Measures***

*Vienna test measures*

Different measures will be collected to compute the cognitive features of the behaviour of road users. The below mentioned measures were already used in earlier phases of the project by partner research institutions.

- *DT* – Vienna Determination Unit (complex reaction tests)
- *MLS* – Motor Performance Series according to Schoppe (fine motor skills)
- *RT* – Vienna Reaction Device (simple reactions tests)
- *ART 90* – Act and React Test System (seven subsets for assessment of fitness to drive)

*Questionnaires*

Before the beginning of the experimental tasks, all participants will fill out a background questionnaire, presented as *Extended data* ^16^, for recruitment purposes and another questionnaire after conducting the respective tasks to reflect on their experiences. This information will be made available to participants before the test and then given to the participants at the end of the experiments in order to fill the form there.

*Simulator data*

The simulator data acquisition system will be used to sample various vehicular data like speed, lateral position, steering wheel angel and braking. Data will be sampled with a frequency of 250 Hz. In total there is one vector per measure for vehicular data: one EEG file containing the signals from the EEG equipment; one vector with the value of NASA-TLX from the questionnaire provided by the driver, including a value before and after the drive; and one vector to track the cognitive load level. Finally, there will be a vector for synchronization with the equipment for physiological measures.

*Physiological measurements*

A number of different physiological data will be collected; heart rate (HR), heart rate variability (HRV), blood volume pulse (BVP), electromyogram (EMG), galvanic skin response (GSR), EEG and electro-oculogram (EOG). For the alcohol test, one additional measurement on BrAC will also be measured. These data will be used to produce model that can give objective measurements of stress and alcohol presence, and as a basis for assessing risky behaviours of road users by corelating with data from the Vienna test and simulators.

***Schedule*.** The tentative time plan for each of the steps for experiments on effects of stress and alcohol on road users are outlined below in Table 8.

**Table 8.** Schedule of various parts of the experiment.

Date	Events
2020-06-01 –	Advertisement for volunteers for stress and alcohol tests
2020-09-16 – 2020-10-31	Information meeting, questionnaire and feedback session for stress and alcohol tests
2020-09-20 – 2020-11-15	Selection of volunteers and signing of consent forms for stress and alcohol tests
2020-10-16 – 2020-11-30	Cognitive assessment by Vienna test of volunteers for stress test
2020-12-01 – 2021-01-15	Cognitive assessment by Vienna test of volunteers for alcohol test
2021-01-15 – 2021-01-31	Pilot study for stress test
2021-02-01 – 2021-02-15	Pilot study for alcohol test
2021-02-16 – 2021-03-31	Main study for stress test with 45 participants
2021-02-20 – 2021-04-05	Self-confrontation interviews for stress test
2021-04-01 – 2021-05-31	Main study for alcohol test with 45 participants
2021-04-16 – 2021-05-15	Self-confrontation interviews for alcohol test
2021-04-16 – 2020-05-20	Miscellaneous tasks

### Data management

Along with the data mentioned above, some personal data - date of birth, sex, weight, height, alcohol drinking habits, driver’s license for cars/motorcycles etc. will be recorded.

***Documentation of investigation procedures*.** The study is an exploratory investigation using the physiological data i.e., the heart (ECG), variation of heartbeat (HRV), cardiovascular dynamics (BVP), skin response (GSR), the muscles (EMG), respiration (RP), activity in the brain (EEG), and the eyes (EOG). The equipment continuously records data from the moment the simulator study is turned on until it is shut down. However, no intervention will be performed and no data regarding intervention will neither be documented nor stored during the data collection.

The alcohol-related tests will be performed with similar physiological data collection routines. In addition, the subject’s reaction to simulated stimuli will be recorded, for example their steering operation and balancing. This will be done by recording speed, lateral position, break and steering wheel angle. The readings of breath alcohol concentration will be performed before and after each recording session.

Video recordings of test participant face will be made to better understand their behaviour during the driving. The road in front of the vehicle will be resumed. Also, data from the simulator i.e., speed, lateral position, break, steering wheel angle, use of the arrows and the behaviour of other vehicles’ drivers will be analysed. Further, there will be audio recording during data collection.

***Data storage and sharing***

*General procedures*

The SIMUSAFE project adopts appropriate procedures so as to ensure the necessary confidentiality and respect of privacy by establishing a set of principles and procedures to guide the partners to achieve the goals and objectives of the SIMUSAFE project. Strict accordance with applicable international, EU and national law, especially the EU directive 95/46/EC is guaranteed, and any related updates will be observed.

The collected data will be handled both locally and in a cloud database. The simulators used for data collection consist of a local server where the initial storage will take place. When a test finishes, data will be de-identified and sent to the IBM’s Cloud storage, where a folder for each test will be created. For data synchronization, Message Queuing Telemetry Transport (MQTT) messages will be used, and data will be encrypted both before transfer to the cloud server and storage in the local server. The GNU Privacy Guard (GPG, also called GnuPG) encryption method will be used for data encryption. The encryption key will only be given to the project partners upon request.

The collected data will be stored for 10 years and will only be used for research and research publication, within the scope of the SIMUSAFE consortium agreement among the partners. The main applicant of this ethical application i.e., Mobyen Uddin Ahmed, will be the responsible person for generating the encrypted code list and the test leaders will have access to the codes.

*General Data Protection Regulation (GDPR) compliance*

SIMUSAFE complies with the recently enacted GDPR ensuring that all personal data collected in the course of this work will follow the letter of the law; for instance, the partners will not collect unnecessary personal data, will keep personal data encrypted or pseudonymized where appropriate, allow respondents to opt out of further processing, to have their personal data erased upon request, and any other data management responsibilities that the GDPR requires the consortium to do. Data are protected according to the Public and Secrecy Act (2009: 400) and the Data Protection Regulation (GDPR).

### Ethical issues

The study plan has been included in an ethical application Dnr: 2019-03961and approved by the Swedish ethical authority Etikprövningsmyndigheten for implementation. During the implementation of the study a study information sheet and a consent form will be provided as presented in as *Extended data* ^16^.

As the methodological approach underlying the SIMUSAFE project relies on experimenting with human beings as a means for the elicitation and reproduction of valid simulated behaviour of artificial actors, ethical issues become a major concern needing appropriate care and attention. Therefore, during the performance of the SIMUSAFE project, ethical issues will be considered during the whole project development, across all working packages. From the initial stage of defining the specifications and requirements for the SIMUSAFE platform, ethical issues management will be defined and any activity involving such actions will be evaluated. A list of several ethical issues is presented in Table 9. This evaluation in particular requires that any actions involving experimentation with human beings and handling sensitive personal information comply with the applicable legal and international scientific standards.

**Table 9.** Several ethical issues are listed considered benefits, effort, risk, and General Data Protection Regulation (GDPR).

Criteria	Descriptions
Benefits for society	Potential reduction of traffic injuries due to improved behaviour as a result of education and training.
Benefits for the company/ university	Taking active part in frontier research. Possible future research and innovation.
Benefits for the test subjects	First-hand knowledge of frontier research, gratification.
Effort for society (EU)	Financial.
Effort for the company/ university	Contributing with research capacity for planning, implementation and analysis. Minimize the risk of unforeseen events or accidents. Insurance.
Effort for the test subjects	Two working days occupied.
Risks for society	Failure to reach research objective, competition from other projects. Unforeseen events.
Risks for the company/ university	Bad reputation in case of failure to meet objectives. Unforeseen events, accidents.
Risks for the test subjects	Accidental person data exposure, other unforeseen events, accidents. After balancing the benefits, efforts, and risks for society, company/university, test subjects, it is concluded that the potential benefits dominate.
GDPR issues	Personal data management. Assignment of responsibility. Right of refusal. Right to terminate participation any time. Right to receive copy of stored personal data. Right to have stored personal data erased. Test person data collection, storage and management.
Code key holder	Mälardalen University (MDH)
Responsible person from MDH	Mobyen Uddin Ahmed
Responsible person from Senseair	Jonas Ljungblad

### Study status

The status of the study is now on hold due to Covid-19 restriction since November 2020. However, there was interest from 127 participants in total and 50 were invited for the information meeting, with 43 having performed the cognitive tests before November 2020.

## Conclusions

This study protocol reports a study on human subjects considering the influence of stress and alcohol on simulated traffic situations. Here, several road users i.e., car, motorcycle, bicycle drivers and pedestrians, are going to be involved in single user and as well as multi-user scenarios. Both control studies with supervision and as well as naturalistic simulation tests are planned. The report also provided a detailed study protocol including test scenarios, study design, platform, data management plan, and related ethical issues. With Covid-19 and the restrictions imposed by the local authority, it has only been permitted to invite the age group 18–24 years for tests, who should not mix with those aged 50–70. As a result, only five participants have been invited as motorcyclists, which has limited the multiuser study to only five set tests. Besides this there have also been some technical issues affecting the study such as problems with video uploading; sometimes the simulator doesn’t upload the video into the Cloud automatically and the problem needs to solve manually. We still hope the Covid-19 restrictions will ease in March/April, and we can invite the older participants to at least perform some more cognitive tests and the stress simulation test.

## Data availability

### Underlying data

No underlying data are associated with this article.

### Extended data

Zenodo: Extended data for a study on human subjects related to Simusafe. https://doi.org/10.5281/zenodo.4836834 ^16^.

This project contains the following extended data within the file ‘Extended data.docx’:

- Information sheet
- Consent forms
- Questionnaire

Data are available under the terms of the Creative Commons Attribution 4.0 International license (CC-BY 4.0).
