# Supplementary material for: Study on human subjects – influence of stress and alcohol in simulated traffic situations
Source: Open Res Eur. 2021 Dec 7;1:83. Originally published 2021 Jul 27. [Version 2] doi: 10.12688/openreseurope.13592.2 (PMC10474340; doi:10.12688/openreseurope.13592.2)
Supplement: Peer review history

# Peer review: Reviewer response for version 2

**DOI:** 10.21956/openreseurope.15408.r33949

**Published:** 2023-09-01

**Authors:** Dario Babic

**Affiliations:** 1 Faculty of Transport and Traffic Sciences, University of Zagreb, Zagreb, Croatia

The report presents a research study plan on the influence of stress and alcohol in simulated traffic situations under an H2020 project named SIMUSAFE. This research study focuses on different road users such as car drivers, motorcyclists, bicyclists and pedestrians. In order to evaluate the influence of stress and alcohol, authors used a range of observatory attributes including physiological measures, simulator data, participants characteristics (e.g., personality, psychological, demographics). The authors described in detailed study design and experimental protocol. Overall, the report is well written and provides good insight into the overall study. I have only two minor comments:

 More detailed comments are presented below:

1. I suggest clearly stating what are “stress” related research questions and what are alcohol related questions. The third research question is not necessary in my opinion.
2. It would be beneficial to have short description of the instruments used for collecting physiological data - the heart and variation of heartbeat, cardiovascular dynamics, skin response, the muscles, respiration, activity in the brain and the eyes.

Is the study design appropriate for the research question?

Yes

Is the rationale for, and objectives of, the study clearly described?

Yes

Are sufficient details of the methods provided to allow replication by others?

Yes

Are the datasets clearly presented in a useable and accessible format?

Yes

Reviewer Expertise:

Driver behaviour

I confirm that I have read this submission and believe that I have an appropriate level of expertise to confirm that it is of an acceptable scientific standard.

# Peer review: Reviewer response for version 1

**DOI:** 10.21956/openreseurope.14662.r27301

**Published:** 2021-08-16

**Authors:** Lior Limonad

**Affiliations:** 1 IBM Research, Haifa, Israel

The paper presents a detailed experimental scheme to explore the potential effects of stress and alcohol consumption on driver’s behavior in a simulator, across a multitude of driver types, including pedestrians. Performance is assessed via a rich set of observatory attributes, including physiological measures, simulator’s state, while also including a rich set of background characteristics about the participants (e.g., personality, psychological, demographics).

 The authors provide a very detailed description of the experimental protocol, one that would allow for subsequent experimental replications in the context of more rigid and confirmatory studies. Overall, the paper is well put, clearly articulated, and recommended for indexing, with only a few minor clarifications and improvement suggestions on my side for its final polishing.

 Hence, my more concrete comments are the following:

- Page 4, end of Background: consider clarifying further why it is important/relevant to study the effect of stress/alcohol in a simulated environment. I am missing something in the spirit of: “Such studies cannot be conducted in realistic conditions, and as such, the simulator provides a faithful alternative, from which many behaviors could be inferred.” And in relation to this, consider also mentioning that this ability may sometimes be shaded by the ‘simulator bias’.
- P4 – “performance of the simulators” -> “performance of drivers’ in simulators”.
- P4 – “in relation to retrospective studies” – stands in conflict with the previous statement of “to our knowledge no studies have been performed”.
- “The following research questions are raised”: consider replacing with “This study is motivated by the following research questions”.
- Regarding the selection of participants, consider adding a clarification onto which population do you wish to draw conclusions about (i.e., generalize to), is it the entire population? of all ages?
- Somewhat related to the previous point – why are you omitting a representation of mid-age 25-49?
- Regarding sample size, what is the basis for choosing a size of 90? Do you have any a-priori knowledge about the foreseen effect size? You may also consider adding a smaller size pilot that is meant to give rationale to sample size.
- “Alcohol usage: 5-15”, why not include also zero to represent someone who doesn’t drink (or rarely drinks)?
- P5 - “machine learning and signal processing”: is it intended to employ supervised / non-supervised learning? if the former, which strategy will be employed for data labeling? if the latter, which techniques may be employed for sufficient data-point population?
- I understand that this is not a confirmatory study, and yet, I would strongly recommend adding a section that states higher-level objectives: i.e., I am missing a general proposition of the following type -
  - “In the study, our aim is to observe [separately] the effects of *Stress*(S) and *Alcohol-consumption*(A) on *driving performance*(P) (i.e., S-->P & A-->P). In the manipulation Stress (excluding pedestrians), we also control for different *Road conditions*(R) that may interfere with the observed effects (i.e., a confounding factor), and include *Situational Risk*(S) as an additional factor that has a potential of interacting with the main effects of stress and alcohol consumption (i.e., S*R*S-->P). b.t.w., this gives rise to the question of why not do the same also with alcohol consumption (i.e., A*R*S-->P). In realization of these, a set of observatory measures are included in the instantiation of each factor as described next ...”
- P6 – “from an actor” -> “from each actor”.
- I think that for most participant types, time pressure (s1) and social pressure (s2) were always manipulated, so for brevity and ease of reading, you can present this before (also with all other factors that may be common) and only list the factors that are different under each specific participant type.
- In the “measures” section, ensure the inclusion of manipulation checking for the main factors manipulated. Consider also adding a measure for the perceived presence of the sensors as causing interaction or distracting drivers from the effect of the intended manipulation of stress and alcohol consumption.
- Also, in relation to the measures section - I strongly recommend moving this section prior to the data analysis/preprocessing section. You may also further partition this. I think that measures here reflect a variety of observable that are split between: 1. measures that could be considered direct instantiation of *road performance* as the main target of the observation, per my understanding, captured mainly via simulator data and physiological measurements). 2. additional background moderators that may later be used to partition the observations (e.g., personality traits, self-perceptions, etc.). I suggest making such a distinction explicit (e.g., as subsections) to clarify how the measures listed correspond to the overall experimental scheme.
- P11 – “de-identifed” -> suggest replacing with 'anonymized'.
- P13 – “set tests” -> “test sets”.
- P13 – “to solve” -> “to be solved”.
- Could also elaborate more on the study establishing a basis for future elaborations such as – interactions between stress and alcohol, between participants (i.e., including multi-participant scenarios), comparisons to realistic settings, etc.

Is the study design appropriate for the research question?

Yes

Is the rationale for, and objectives of, the study clearly described?

Yes

Are sufficient details of the methods provided to allow replication by others?

Yes

Are the datasets clearly presented in a useable and accessible format?

Yes

Reviewer Expertise:

Human-centric AI

I confirm that I have read this submission and believe that I have an appropriate level of expertise to confirm that it is of an acceptable scientific standard.
